# Investigation of the role of polymorphic variants FTO gene in schizophrenia patients with metabolic syndrome

**DOI:** 10.1192/j.eurpsy.2021.1028

**Published:** 2021-08-13

**Authors:** I. Pozhidaev, D. Paderina, A. Boiko

**Affiliations:** Mental Health Research Institute, Tomsk National Research Medical Center of Russian Academy of Sciences, Tomsk, Russian Federation

**Keywords:** Metabolic syndrome, polymorpism, FTO gene, schizophrénia

## Abstract

**Introduction:**

Medication is primary tactics in schizophrenia treatment. First and second generation antipsychotics (FGA and SGA respectively) affect on core symptoms. Unfortunately, it causes side effects. Metabolic syndrome one of them and includes large number of affections like body mass index changes, lipidemias, hypertension and others.

**Objectives:**

To study the role of polymorphic variants FTO gene in metabolic syndrome in schizophrenia patients.

**Methods:**

We were investigated 480 patients. Main criteria for inclusion in study was using antipsychotics, verified diagnosis of schizophrenia and metabolic syndrome. Mean age was 42,1±1,4 years. The metabolic syndrome was assessment based on clinical data. Standard phenol-chloroform protocol for DNA isolation was used. Genotyping was carried out on six SNP’s of FTO gene with real-time PCR. Statistical analysis was carried out with R 3.6.2 with basic functions and SNPassoc package.

**Results:**

The distribution of genotypes for variants rs7185735 and rs9939609 was not in according to Hardy-Weinberg equilibrium (a p-value less than 0.05) and excluded from further analysis. Patients with schizophrenia were divided into two groups: patients with metabolic syndrome and patients without it. We did not identify any statistically significant associations between genotypes and alleles of FTO gene and metabolic syndrome.

**Conclusions:**

We did not find any associations of alleles and genotypes of FTO gene with metabolic syndrome in schizophrenia patients from Siberia region. Metabolic syndrome needs more further studies with larger number of samples and different populations. Conflict of interest. The authors declare no conflict of interests. Supported by Grant of RSF 19-75-10012.

